# Deep centroid: a general deep cascade classifier for biomedical omics data classification

**DOI:** 10.1093/bioinformatics/btae039

**Published:** 2024-02-01

**Authors:** Kuan Xie, Yuying Hou, Xionghui Zhou

**Affiliations:** Hubei Key Laboratory of Agricultural Bioinformatics, College of Informatics, Huazhong Agricultural University, Wuhan 430070, People’s Republic of China; Hubei Key Laboratory of Agricultural Bioinformatics, College of Informatics, Huazhong Agricultural University, Wuhan 430070, People’s Republic of China; Hubei Key Laboratory of Agricultural Bioinformatics, College of Informatics, Huazhong Agricultural University, Wuhan 430070, People’s Republic of China; Key Laboratory of Smart Farming for Agricultural Animals, Huazhong Agricultural University, Wuhan 430070, People’s Republic of China

## Abstract

**Motivation:**

Classification of samples using biomedical omics data is a widely used method in biomedical research. However, these datasets often possess challenging characteristics, including high dimensionality, limited sample sizes, and inherent biases across diverse sources. These factors limit the performance of traditional machine learning models, particularly when applied to independent datasets.

**Results:**

To address these challenges, we propose a novel classifier, Deep Centroid, which combines the stability of the nearest centroid classifier and the strong fitting ability of the deep cascade strategy. Deep Centroid is an ensemble learning method with a multi-layer cascade structure, consisting of feature scanning and cascade learning stages that can dynamically adjust the training scale. We apply Deep Centroid to three precision medicine applications—cancer early diagnosis, cancer prognosis, and drug sensitivity prediction—using cell-free DNA fragmentations, gene expression profiles, and DNA methylation data. Experimental results demonstrate that Deep Centroid outperforms six traditional machine learning models in all three applications, showcasing its potential in biological omics data classification. Furthermore, functional annotations reveal that the features scanned by the model exhibit biological significance, indicating its interpretability from a biological perspective. Our findings underscore the promising application of Deep Centroid in the classification of biomedical omics data, particularly in the field of precision medicine.

**Availability and implementation:**

Deep Centroid is available at both github (github.com/xiexiexiekuan/DeepCentroid) and Figshare (https://figshare.com/articles/software/Deep_Centroid_A_General_Deep_Cascade_Classifier_for_Biomedical_Omics_Data_Classification/24993516).

## 1 Introduction

The rapid advancement of next-generation sequencing technologies has led to the generation of large-scale omics data, providing ample opportunities for harnessing machine learning models to investigate associations between genetic features and specific phenotypes, such as cancer, as well as predicting sample categories based on omics features. However, omics data often possess high-dimensional features and are constrained by limited sample sizes (Sun *et al.* 2019, Basavegowda and Dagnew 2020). Furthermore, data from different sources often exhibit inherent inconsistencies that are difficult to eliminate (Papiez *et al.* 2019). Consequently, these factors pose significant hurdles in constructing classifiers with exceptional classification performance and robust discriminative ability across datasets derived from various sources (Demšar and Zupan 2021, Greener *et al.* 2022).

In recent years, machine learning, especially deep learning, has made significant progress in various fields, and deep learning models have achieved remarkable breakthroughs in some biomedical domains (Jumper *et al.* 2021, Townshend *et al.* 2021, Baek and Baker 2022, Cheng *et al.* 2023). Due to the typically large-scale training data required for deep learning models, deep learning has achieved remarkable success primarily in tasks such as molecular structure prediction, where abundant training data are available. However, for most biomedical omics data, characterized by small sample sizes and high data dimensions, there is currently no widely accepted universal deep learning model suitable for omics classification problems. It is worth noting that many deep learning models suffer from limited interpretability, a concern that aligns with the biological functional relevance of features often emphasized in biomedical omics research, further constraining the applicability of deep learning models in this domain.

To address the issues of large training sample requirements and lack of interpretability in deep learning models, Zhou and colleagues (Zhou and Feng 2017, Zhou and Feng 2019) developed a cascaded classifier based on random forest called Deep Forest (DF). This model has achieved performance comparable to deep learning models on multiple datasets. Some researchers have modified the DF architecture to make it suitable for biomedical data (Su *et al.* 2019, Chu *et al.* 2021, Wu *et al.* 2023). However, these studies still lack validation on independent datasets and are only applicable to specific problems. Therefore, an easy-to-use, general, and robust classification model is crucial for the application of biomedical omics data.

The centroid classifier (Simon 2003), also known as Nearest Centroid Classifier, is renowned for its simplicity and consistent performance on independent datasets (Lu and Zhou 2019). Simultaneously, the deep cascade strategy significantly enhances the model's fitting capability. Building upon these strengths, we present Deep Centroid, a novel classifier that combines the stability of the centroid classifier with the robust fitting ability of the deep cascade strategy. Deep Centroid uses an ensemble learning approach with a multi-layer cascade structure, comprising feature scanning and cascade learning stages that allow for dynamic adjustment of the training scale. To validate the effectiveness of our model, we applied Deep Centroid to three important problems in precision medicine research: early cancer diagnosis, cancer prognosis, and drug sensitivity prediction, utilizing diverse biomedical omics data as features. The results demonstrate that our model outperforms six alternative models in both cross-validation and independent validation. Notably, the features identified by Deep Centroid hold biological significance, highlighting the potential applicability of our approach. In addition, we provide a comprehensive Python package for Deep Centroid, equipped with user-friendly functions and interfaces for parameter tuning. This package ensures effective and robust classification using biomedical omics data.

## 2 Materials and methods

### 2.1 Datasets

Our method has been applied to three typical precision medicine domains: early cancer diagnosis, cancer prognosis, and drug sensitivity prediction. For early cancer diagnosis, we obtained whole-genome cell-free DNA (cfDNA) sequencing data from plasma samples of lung cancer patients, referred to as the LUCAS dataset and the LUCAS validation dataset, from Dimitrios *et al.*'s paper (Mathios *et al.* 2021). Following the approach outlined by Ulz *et al.* (2016), we used the number of fragments near the transcription start site (TSS) within [−50, 150] as features to construct the early cancer diagnosis model. Consistent with Dimitrios *et al.*'s approach, we used the LUCAS dataset for cross-validation and model construction and the LUCAS validation dataset for independent validation. In the case of cancer prognosis, we utilized four breast cancer transcriptome datasets along with clinical information for each sample. Building upon our previous work (Lu and Zhou 2019), we used the GSE2034 dataset (Wang *et al.* 2005) for cross-validation and model construction, while the union of the other three datasets (Desmedt *et al.* 2007, Schmidt *et al.* 2008, Zhang *et al.* 2009) was used for independent validation. All transcriptome data were based on the GPL96 chip, and chip probe information was mapped to genes. For each gene, the average value of all probes was used as the gene's value. For each patient, if the metastasis/recurrence event occurred within five years, they were considered as samples with poor prognosis. If a patient's event-free survival exceeded five years, they were considered to have a good prognosis, and other samples were discarded. All datasets for drug sensitivity prediction were obtained from GDSC (Genomics of Drug Sensitivity in Cancer) (Iorio *et al.* 2016), and we used 949 cell line datasets that included DNA methylation data (GSE68379) and gene transcriptome data (RMA normalized expression data for cell lines). The drug response information for 48 FDA-approved drugs in these cell lines was used as class labels. Since there was no independent validation dataset, cross-validation was used to evaluate our models for each drug. For all the three tasks, *z*-score was used to normalize the datasets. The details of the datasets were shown in Table 1.

**Table 1.** Details of all the datasets used in this work.

Dataset name	Number of samples	Sample ratio (positive versus negative)	Application
LUCAS	287	1:1.22	Early detection
LUCAS validation cohort	431	1:8.37	
GSE2034	276	1:1.97	Cancer prognosis
GSE7390	190	1:4.28	
GSE11121	182	1:5.50	
GSE12093	136	1:10.33	
GSE68379	949 cell lines	/	Drug sensitivity prediction
Expression data	949 cell lines	/	
S5C	48 drugs on 949 cell lines	1:7.65	

### 2.2 Deep centroid classifier

Deep Centroid Classifier is a deep cascaded ensemble classifier that utilizes centroid classifier (Supplementary Note S1) as the base classifier. It takes heterogeneous omics data from one or multiple layers of samples as input and outputs binary class labels or scores for the samples. The model consists of three stages: feature scanning, deep cascading, and majority voting.

#### 2.2.1 Feature scanning

In the field of image processing, local information in images is beneficial for subsequent recognition tasks. Therefore, convolutional neural networks utilize convolutional kernels to extract meaningful local information for further analysis. However, in omics data, gene features are usually arranged in the order of names or IDs, as a result, adjacent features in the data often lack biological correlation. In this paper, we use many random scans to obtain feature combinations involved in certain biological functions (such as biological processes). The number of random scans and the size of each random feature sets can be adjusted based on the number of candidate features (see Supplementary Fig. S1 for the scan methods). Our toolkit also provides parameter structures for user selection. In addition, we offer known functional gene sets (Go Term biological process and KEGG pathways from MSIGDB) as candidate feature sets, which can be selected by the users through parameters. In the meanwhile, the model adds a known feature set interface, which can be imported by the users for known feature sets to provide some prior knowledge for feature scanning.

#### 2.2.2 Cascading learning

Deep Centroid utilizes a multi-layer cascade structure to construct the model, where multiple centroid classifiers are trained separately in each layer. The output results, along with the scanned initial data, are then used as input for the subsequent layer until the model achieves convergence. In addition, the model has a pruning function, which will delete classifiers with low scores to improve prediction accuracy. The scanned initial features are sets obtained using the method described in the feature scanning stage. Each set is trained with a machine learning model and scored to generate a new feature. Within the package, users have the flexibility to choose different base classifiers, such as centroid classifier (default), Support Vector Machine (SVM), Random Forest (RF), and more. By incorporating spliced data, the training dataset can be supplemented to avoid insufficient training features or highly concentrated data features that may result in rapid model convergence. This approach enhances the reusability of the original data and improves the model's fitting ability. The details of the cascading learning strategy are shown as follow:

1. In the training set, a re-sampling strategy (sampling with replacement) is used to ensure that the samples involved in training the classifiers have relatively balanced categories. The sampling ratio of the two categories is defined by sampling coefficient (adjustable, default: 0.65). The samples that are not included in the sampling process are used as the validation set to evaluate the performance of the model.
2. The samples used for training the model are divided into two classes by the centroid classifier based on their labels. For each class, the centroid vectors are computed for each feature, resulting in n sets of centroid vectors. Each set contains both positive and negative centroid vectors.
3. The validation set is used to evaluate the classification performance, and classifiers with MCC ≤ Threshold (adjustable, default: 0) are removed. The sample score is obtained by calculating the distance between the sample and the centroid vector.
4. If the classification performance of the current layer improves compared to the previous layer, training continues to the next layer. The n-centroid distances output by the n-centroid classifiers are used as new data features, which are combined with the scanned features from the original data (or the other types of omics data) and serve as input for the next layer.
5. Training is stopped if the classification performance of the current layer no longer improves.

#### 2.2.3 Majority voting

When the classification performance of the model no longer improves, the model ceases to cascade further and obtains the final prediction for each sample by majority voting among all classifiers in the last layer. Depending on user-defined parameters, in addition to the predicted class labels for the samples, the model can also provide the probability values indicating the likelihood of the samples belonging to the positive class.

#### 2.2.4 Data fusion strategy

Our model is designed to handle both single-level omics data and multi-omics data. For single omics data, this data serves as the input to the data input layer, and after undergoing feature random scanning, it is used as the input to the Nearest Centroid Classifier (NCC). Simultaneously, to prevent rapid convergence during cascading, the omics data, after feature scanning, is used as new information for each cascaded layer. For multi-omics data, multiple omics data are concatenated and used together as the input for the first layer and subsequent deep cascaded layers.

### 2.3 Evaluations

To evaluate the performance of Deep Centroid (DC), we compared it with six classical classifier models: Rand Forest (RF) (Ho 1995), Support Vector Machine (SVM) (Cortes and Vapnik 1995), Deep Forest (DF) (Zhou and Feng 2017), eXtreme Gradient Boosting (XGBoost) (Chen and Guestrin 2016), Nearest Centroid Classifier (NCC) (Hart *et al.* 2000, Blagus and Lusa 2013), and Deep Neural Network (DNN) (Liu *et al.* 2021). For early cancer diagnosis and cancer prognosis analysis, we performed 10 times 5-fold cross-validation on one dataset and conducted independent validation on another dataset (for cancer prognosis, the merged set of three different datasets were used), as detailed in Table 1. For drug sensitivity prediction, due to the lack of an independent dataset, we presented the average results of 10 times 5-fold cross-validation for 48 clinically approved drugs. As many models, including our Deep Centroid classifier, used random seeds during training, the independent validation results presented are the averages of multiple independent validations. For more comprehensive information regarding the specific experimental parameters used for each model, please refer to Supplementary Note S2.

We evaluated the performance of each classifier using metrics such as Matthews Correlation Coefficient (MCC), Area under the curve (AUC), Accuracy, and F1-score. Since omics data often face the issue of class imbalance, this study primarily utilized MCC, a metric that effectively evaluates classification performance on imbalanced datasets, as the main evaluation criterion. For the details of all the indices, please refer to Supplementary Note S3.

### 2.4 Ablation experiments

Our model innovates in feature scanning and base classifier selection compared to Deep Forest. To confirm these enhancements' impact on performance, we performed two ablation experiments.

#### 2.4.1 Feature scanning ablation experiment

In the feature scanning ablation experiment, we compared two strategies for feature selection: random scanning and sliding window scanning. For random scanning, we randomly selected feature sets of varying sizes (from 10 to 200 features) from the original feature set. The features within each set were chosen randomly. On the other hand, sliding window scanning involved selecting contiguous sets of neighboring features from the original features. In our ablation implementation, the size of the sliding window feature sets was fixed at 100, corresponding to the median size of the random sets. In addition, the step size for sliding the window was set to *X* (defined as the total number of features divided by the number of feature sets). Both random scanning and sliding window scanning resulted in a total of 500 feature sets.

#### 2.4.2 Base classifier ablation experiment

In Deep Centroid, the nearest centroid classifiers were used as base classifiers. In the ablation experiments, we compared this strategy with two others: random forest (denoted as Integrated RF) and multiple models (denoted as Integrated MM). For Integrated RF, all NCC were replaced with RF. For Integrated MM, we kept the centroid classifiers as the first layer of the model. In each layer after the first layer, we used five types of classifiers—NCC, RF, support vector machines (SVM), XGBoost, and deep neural networks (DNN)—as the base classifiers. The parameters of all classifiers were consistent with the previous description.

## 3 Results

In the results section, we first provide a brief overview of the method's framework. Subsequently, we conducted a comprehensive assessment of Deep Centroid in three critical domains: early cancer diagnosis, cancer prognosis, and drug sensitivity prediction. This included a comparison of its classification performance with six typical, general-purpose classifiers. We also performed functional interpretation of the features identified by the DC model to demonstrate the biological interpretability of our model. Finally, we carried out ablation experiments on the two key innovations of Deep Centroid, namely using centroid classifiers as base classifiers and implementing random feature scanning, to clarify the significance of these modifications in Deep Centroid.

### 3.1 The mainframe of deep centroid

**Figure 1.**
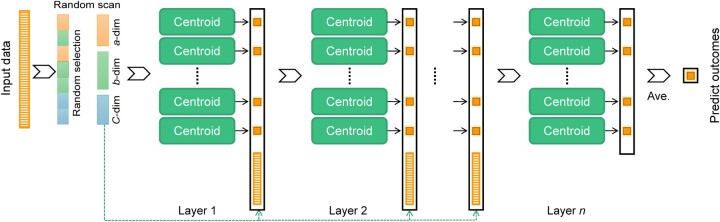
Schematic diagram of the model structure of Deep Centroid. In the feature scanning stage, using random scanning strategy, the model divides heterogeneous data into multiple feature sets, with each feature set corresponding to one nearest centroid classifier. After each layer of the model in the cascade learning stage is trained, the output results are integrated with the optimized features as new features and continue to be used for the next layer of training. When the model converges, the model stops training and uses the majority votes to obtain the predicted result.

Considering the robustness of the centroid classifier and the strong fitting ability of the deep cascading strategy, we introduce a novel deep cascading classifier, the Deep Centroid classifier (Fig. 1). Deep Centroid consists of three stages: feature scanning, deep cascading, and result prediction. In the feature scanning stage, considering that the feature arrangement in biomedical data has no actual biological significance, local scanning cannot extract meaningful local features like image data. Therefore, we use a random scanning method (by default, the strategy used in this manuscript) and an optional feature scanning strategy that utilizes biological prior knowledge (biological functional gene sets) to extract features from high-dimensional and heterogeneous input data. In the deep cascade stage, each layer uses several centroid classifiers as base classifiers. The first-layer centroid classifiers receive features extracted by the feature scanning stage as input, after centroid classifier calculations, and the results are used as input for the second-layer centroid classifiers. Subsequent layers receive inputs from the centroid classifiers of the previous layer and provide input to the next layer. Meanwhile, to prevent the deep cascade process from converging too quickly, each cascade layer also receives features scanned during the feature scanning stage to provide new information to the cascade layers. In the prediction result stage, we use majority voting to calculate the score and corresponding label for each sample. Please refer to the Method section for detailed steps of the Deep Centroid classifier.

### 3.2 Application of deep centroid to cancer early detection

**Figure 2.**
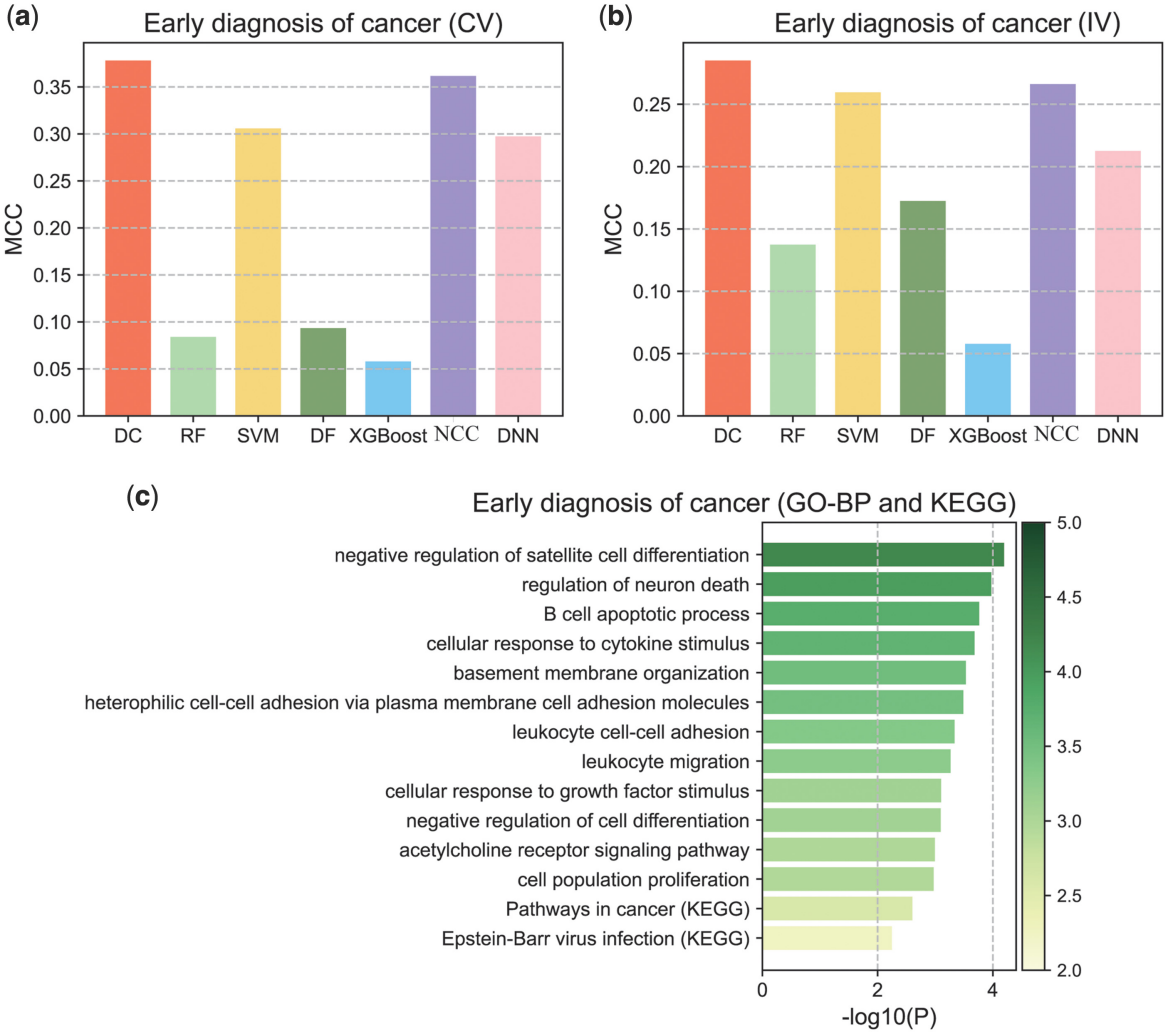
Performance of Deep Centroid in cancer early detection. (a) Classification performance of Deep Centroid in cross-validation. (b) Classification performance of Deep Centroid in independent validation. (c) Functional annotation results of important features.

Early cancer diagnosis can significantly reduce the mortality rate among cancer patients, and cell-free DNA fragmentomics data has proven to be an effective, minimally invasive biomarker for early cancer diagnosis (Mathios *et al.* 2021). We obtained whole-genome cfDNA sequencing data from lung cancer patients and controls from Dimitrios *et al.*'s paper (LUCAS dataset and LUCAS validation dataset, Table 1) and calculated the number of fragments near transcription start sites as features (Ulz *et al.* 2016). Following the same strategy in the original paper (Mathios *et al.* 2021), we used the LUCAS dataset for cross-validation and model construction, with the LUCAS validation dataset serving as an independent test set. In a comparative analysis with six other methods (Fig. 2a and b, Supplementary Tables S1 and S2), our method outperformed the others in both cross-validation and independent validation. Apart from our method, the Nearest Centroid classifier and SVM also demonstrated good performance, indicating that in such high-dimensional, low-sample datasets, simpler models may yield more stable classification abilities. In addition, our deep cascading strategy indeed improved the fitting ability of the Nearest Centroid classifier.

To evaluate whether our feature scanning strategy identified important genes for cancer diagnosis, we selected the important features scanned by our method (genes included in base classifiers with high classification performance, detailed methods in Supplementary Note S4) and performed an enrichment analysis on these features (Supplementary Note S5). All enrichment results are provided in Supplementary Table S3, with key results presented in Fig. 2c. The results indicate that the features scanned by our strategy were primarily enriched in cancer-related pathways (such as Pathways in Cancer), functional gene sets related to cell differentiation, apoptosis, and cell adhesion. In addition, some pathways were enriched in immune cell or other leukocyte-related pathways. As we know, cfDNA in plasma mainly originates from white blood cells (Bryzgunova *et al.* 2021), and these annotation results validate that our feature scanning strategy can indeed identify features of significant biological relevance, further affirming the reliability of our model.

### 3.3 Application of deep centroid to cancer prognosis

**Figure 3.**
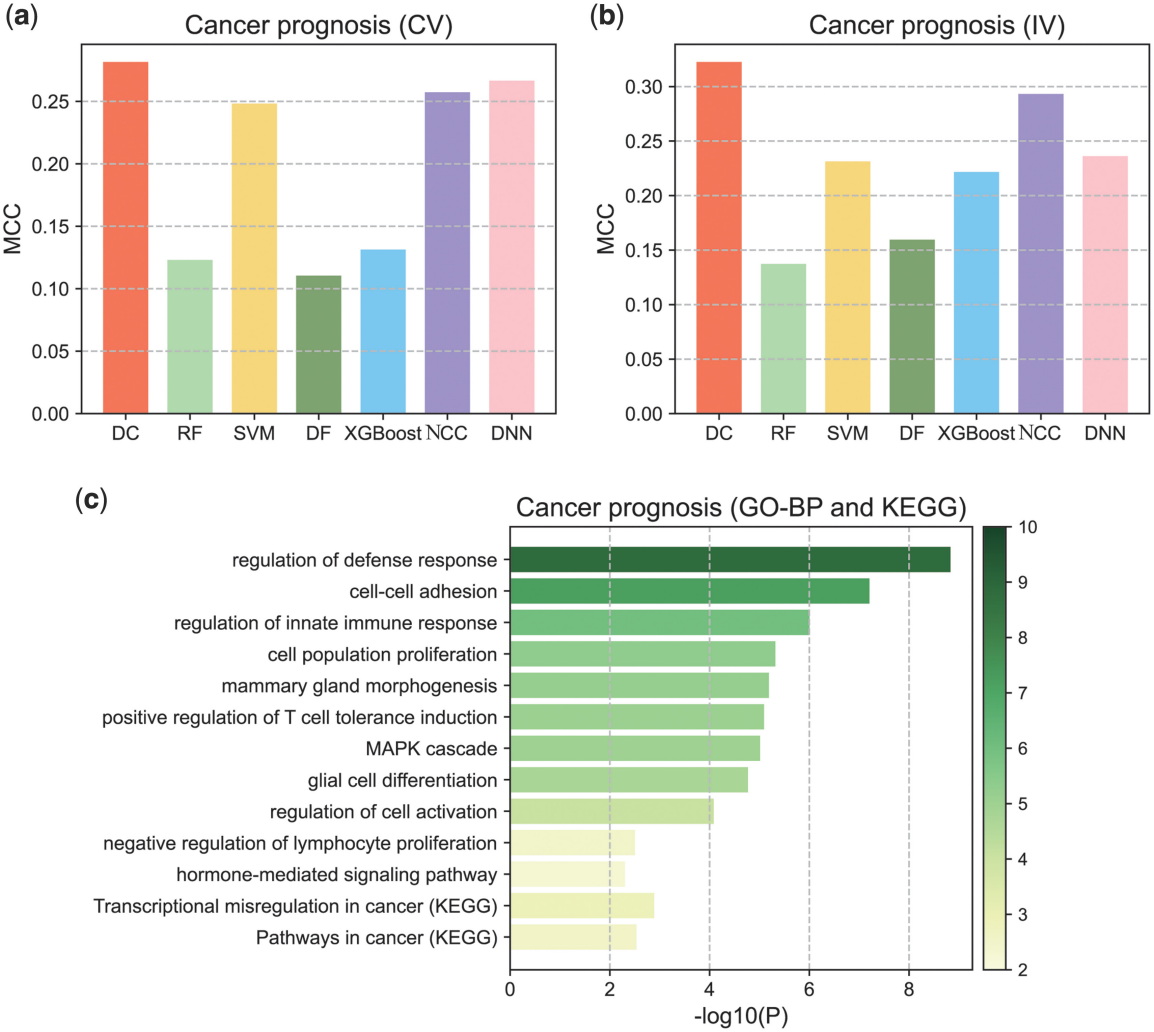
Performance of Deep Centroid in cancer prognosis. (a) Classification performance of Deep Centroid in cross-validation. (b) Classification performance of Deep Centroid in independent validation. (c) Functional annotation results of important features.

Cancer prognosis plays a guiding role in the treatment of cancer patients (Wang *et al.* 2005). We downloaded the transcriptome data and corresponding prognosis information of four breast cancer patients from NCBI GEO, and selected the dataset with the largest sample size, GSE2034 (Wang *et al.* 2005), to build the model and perform cross-validation, while using the other three datasets as independent datasets. As shown in Fig. 3a and b (see Supplementary Tables S4 and S5 for detailed results), our model performs better in both cross-validation and independent validation, followed by Centroid classifier, SVM, and DNN.

We also performed functional annotation on the important features identified from the breast cancer prognosis dataset, as shown in Fig. 3c and Supplementary Table S6. Theenriched functional gene sets include Pathways in Cancer, cell differentiation, cell adhesion, immune response, and other cancer-related functions. Notably, the annotation results also include hormone-mediated signaling pathway and mammary gland morphogenesis, indicating that our feature scanning strategy can identify genes highly relevant to breast cancer development and prognosis.

### 3.4 Application of deep centroid to drug sensitivity prediction

**Figure 4.**
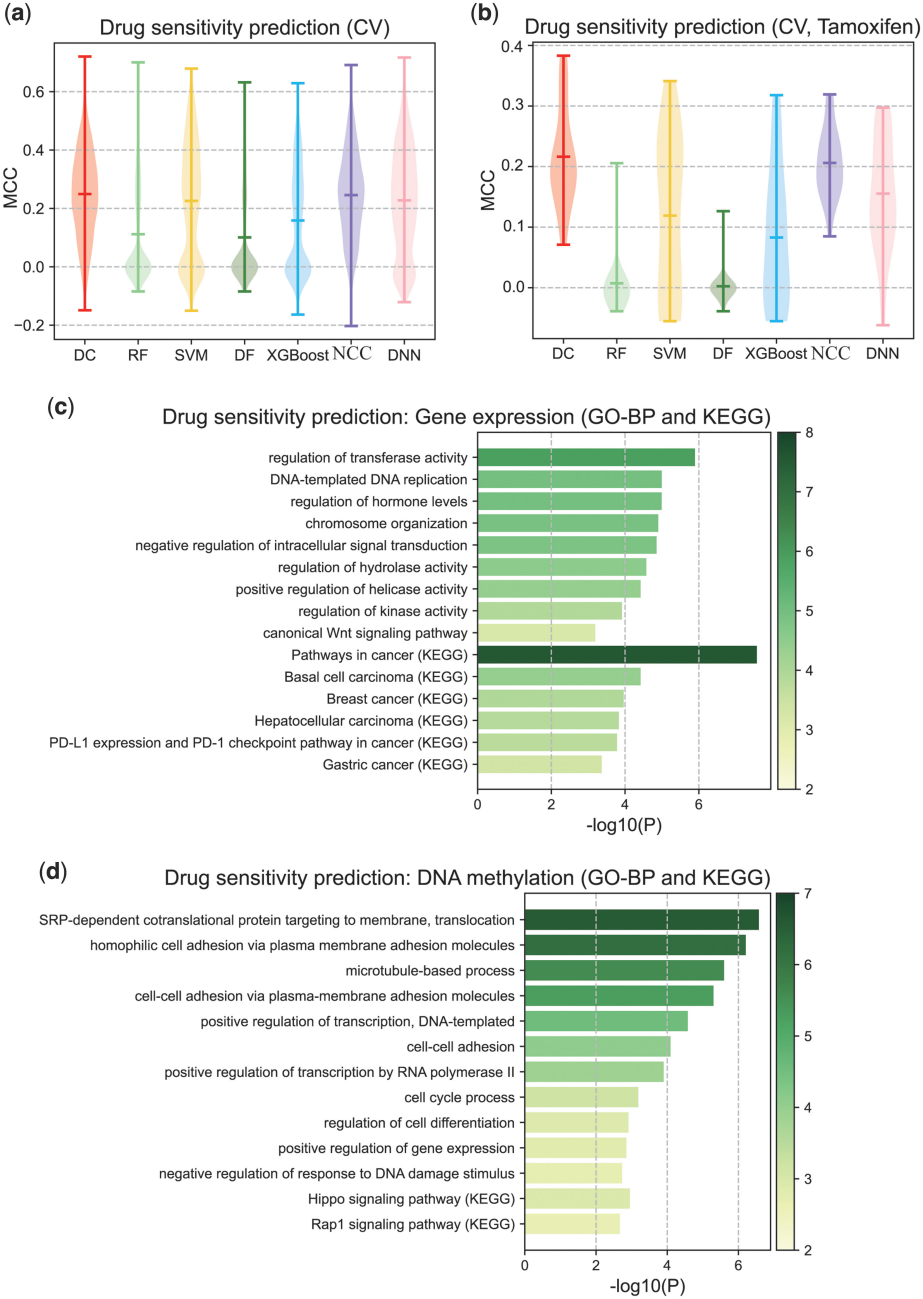
Performance of Deep Centroid in drug sensitivity prediction. (a) Classification performance of Deep Centroid in all the drugs. (b) Classification performance of Deep Centroid in Tamoxifen. (c) Functional annotation results of important features scanned in gene expression data. (d) Functional annotation results of important features scanned in DNA methylation data.

We used 949 cells from the CCLE dataset that have gene expression data, DNA methylation data, and information on whether they are sensitive to 48 FDA-approved drugs to test the performance of the Deep Centroid classifier in predicting drug sensitivity. We built a model for each cell line to predict whether it is sensitive or resistant to each drug, using cross-validation to evaluate our models. In each model, the cell line's gene expression data was used as input features for the Deep Centroid classifier, while the cell line's DNA methylation scan results were used as additional features for each subsequent cascade layer to avoid premature convergence. The overall results of the 48 models are shown in Fig. 4a (Supplementary Table S7). The results show that the Deep Centroid Classifier performed best, followed by NCC, SVM, and DCC. In addition, we conducted a case study of the famous anticancer drug Tamoxifen and found that its results were similar to the overall results, with our method still performing best (Fig. 4b).

Finally, we conducted functional annotation on the features identified by the Tamoxifen model, which included both gene expression and DNA methylation data. We analyzed the important features identified in gene expression data and DNA methylation data separately (Fig. 4c and Supplementary Table S8 for gene expression data, Fig. 4d and Supplementary Table S9 for DNA methylation data). The results show that these features are enriched not only in pathways in cancer, cell adhesion, DNA replication, cell differentiation, cell cycle, DNA damage repair, and PD1 (PDL1) related biological process but also in breast cancer-specific functions such as regulation of hormone level and ‘breast cancer’. In the meanwhile, Tamoxifen is a drug used for breast cancer treatment (Osborne 1998). In addition, these features are also enriched in biological functions related to drug transport and metabolism, such as regulation of transferase activity. All these results suggest that our model not only has stable classification performance but also has biologically interpretable results.

### 3.5 Ablation experiments

We conducted ablation studies to assess the impact of two key components of our model: the feature scanning strategy and the selection of the NCC as the base classifier. In terms of the feature scanning strategy, we compared our approach (random scanning) with method used by Deep Forest (sliding window scanning). Regarding the base classifiers, we compared our approach with two different methods: Random Forest as the base classifier (denoted as Integrated RF) and several models as base classifiers (denoted as Integrated MM, Method).

**Figure 5.**
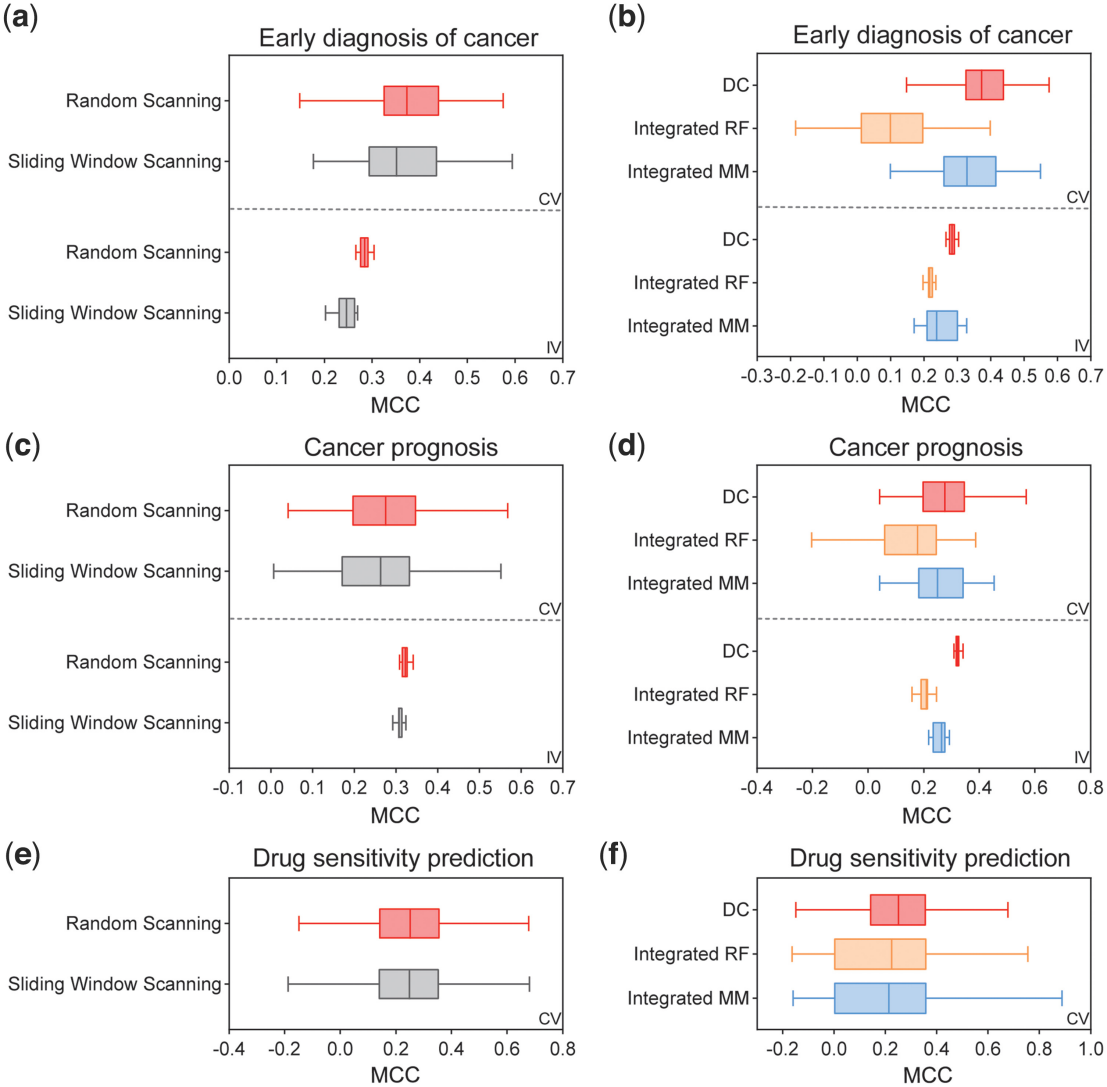
Ablation experiment results. Performance comparison between models using random scanning strategy and sliding window scanning strategy in early cancer diagnosis (a), cancer prognosis (c), and drug sensitivity prediction (e). Performance comparison between models using nearest centroid classifier as the base classifier and models applying random forest as the base classifier, as well as the model applying multiple classifier models as the base classifier in early cancer diagnosis (b), cancer prognosis (d), and drug sensitivity prediction (f).

The results of the ablation experiments in terms of cross-validation and independent validation for cancer early detection and cancer prognosis, as well as cross-validation for drug sensitivity prediction, are presented in Fig. 5. These results clearly demonstrate that both contributions made by Deep Centroid significantly enhance the model's performance (see Supplementary Tables S10–S15 for detailed information).

## 4 Conclusion and discussion

With the advancement of sequencing technologies, an increasing number of omics resources (Kaushik *et al.* 2020a,b) and methods (Kaushik *et al.* 2020a,b; Zhao *et al.* 2023) have been developed to address biomedical challenges. Sample classification based on biological omics data is a common task in biomedical research. However, due to the high feature dimension, low sample size, and the insufficient reproducibility of data from different sources, although classifier models have achieved great success in other fields, there is still a lack of stable and reliable general classifier models for sample classification of biological omics data. Leveraging the stability of nearest centroid classifier and the strong fitting ability of the deep cascading strategy, we propose a novel deep cascading ensemble model. In this model, during the feature scanning stage, we use a random scanning strategy (while also providing a biological prior knowledge-based scanning strategy for user selection) to extract biologically meaningful feature sets. In the deep cascading stage, we use nearest centroid classifiers as base classifiers. In the final prediction stage, we apply a majority voting strategy. This model has achieved better results than mainstream classification models in three typical applications of precision medicine: early cancer diagnosis (cfDNA fragmentomics data), cancer prognosis (gene transcriptome data), and drug sensitivity prediction (gene transcriptome data and DNA methylation data). In addition, the model's feature scanning stage can scan biologically meaningful essential features.

We found that Deep Centroid had the best performance in the MCC evaluation metric for predicting class labels, and performed well in AUC, but not always the best. This may be due to the primary advantage of nearest centroid classifiers, which are simple, resistant to overfitting, and exhibit stable predictive performance. This characteristic allows our model to predict sample class labels quite accurately across different datasets, consistently resulting in a high MCC metric. However, the drawback of NCC is its limited fitting ability. Although our deep cascaded strategy enhances the model's fitting ability (our model consistently outperforms a simple NCC), it is constrained by NCC's fitting ability, resulting in our model showing the best performance in MCC but not always in AUC. As far as we know, providing a precise class category for a sample (rather than its score for belonging to a certain class) is more useful for practical clinical applications. Therefore, we believe our model holds significant value. Of course, exploring how to further improve the model's fitting ability based on NCC will be a focus of our future work, specifically in the deep cascading stage to provide more diverse information to prevent nearest centroid classifiers from being overly stable and unchanging, resulting in faster convergence without further enhancement of the model's fitting ability.

In addition, all three applications of our model have high-dimensional and low-sample-size data, so the results only show that our method can be well applied to sample classification of typical biological omics data. In other biomedical research applications such as predicting protein structure, predicting genomic variant sites, and chromatin open regions, the application value of our model still needs further validation. The issues will be the focus of our future work.

Anyway, we have proposed a general ensemble classification model with stable classification capabilities for sample classification of biological omics data in biomedical research. This model not only has good classification performance but also has biological interpretability. In addition, we have created a user-friendly Python toolkit for this model, providing valuable support for biomedical research focused on biological omics data analysis.

## Supplementary Material

btae039_Supplementary_Data
