# Supplementary material for: Deep centroid: a general deep cascade classifier for biomedical omics data classification
Source: Bioinformatics. 2024 Feb 1;40(2):btae039. doi: 10.1093/bioinformatics/btae039 (PMC10868341; doi:10.1093/bioinformatics/btae039)
Supplement: btae039_Supplementary_Data [file btae039_supplementary_data.docx]

**SUPPLEMENTARY INFORMATION FOR**

**Deep Centroid: A General Deep Cascade Classifier for**

**Biomedical Omics Data Classification**

Kuan Xie^1^, Yuying Hou^1^, Xionghui Zhou^1,2*^

^1^Hubei Key Laboratory of Agricultural Bioinformatics, College of Informatics, Huazhong Agricultural University, Wuhan, 430070 People’s Republic of China

^2^Key Laboratory of Smart Farming for Agricultural Animals, Ministry of Agriculture and Rural Affairs, People’s Republic of China

^*^To whom correspondence should be addressed. (zhouxionghui@mail.hzau.edu.cn; zhouxionghui6@gmail.com)

**Supplementary Information includes:**

Supplementary Notes 1 to 5.

Supplementary Figures 1.

Supplementary Tables 1 to 15.

Supplementary References.

**Supplementary Note 1: The details of Nearest Centroid classifier**

Suppose there is a set N of *n* cancer patients: i_1_, i_2_, ..., i*_n_*, and a set M of *m* healthy individuals: j_1_, j_2_, ..., j*_m_*. Taking the gene expression profile as an example, the total number of features is *T*. The feature *q* of sample S*_p_* on the gene expression profile is described as the expression of that gene or tissue under specific conditions, which is measured as *Express*(*p*, *q*) where 0≤*p*<(*n*+*m*), 0≤*q*<*T*. Then, the centroid corresponding to the feature *q* of cancer patients can be calculated according to Equation (1), which is the mean value of the expression values of all cancer samples corresponding to this feature. Similarly, the centroid corresponding to the feature *q* of healthy individuals can be calculated according to equation (2).

$C_{q}^{+}=\frac{1}{n}\sum_{Sp\in N} Express\left( p, q \right)$ (1)

$C_{q}^{-}=\frac{1}{m}\sum_{Sp\in M} Express\left( p,q \right)$ (2)

The *T*-dimensional centroid vector $\vec{C}^{+}$ of the positive samples can be obtained from all the feature centroids of the cancer patient group, and the *T*-dimensional centroid vector $\vec{C}^{-}$ of the negative samples corresponds to all the feature centroids of the healthy person group. Next, the vector mean and vector weight of the two vectors can be calculated, as shown in Equation (3) and Equation (4).

$\vec{C}=\left( \vec{C}^{+}+\vec{C}^{-} \right)/2$ (3)

$\vec{W}=\vec{C}^{+}-\vec{C}^{-}$ (4)

For a new patient sample *s* = f_1_, f_2_, ..., f*_T_*, *T* is the total number of features. The centroid distance between this sample and the training sample can be calculated, as shown in Equation (5).

$distance=<s-\vec{C}, \vec{W}>$ (5)

**Supplementary Note 2: Implementations of all the classifiers**

DeepCentroid: The number of feature sets is 500 (default number). The number of feature sets in cancer diagnosis is set as 1000 because the number of features in cell-free DNA data is large. The feature set size is [10, 200], and the sampling coefficient is 0.65 in all tasks. With the provided interfaces of the Python package, users can easily configure the aforementioned parameters. If the users does not make any settings, the package provides default values.

RF: The number of decision trees is 100, and the feature measurement standard is *gini* coefficient.

SVM: The regularization coefficient is 10, the kernel function is *rbf*, and probability output is enabled.

DF: Two types of RF, each with 2 and containing 100 trees. The characteristic measurement standard is *gini* coefficient, and the number of data buckets is 255.

XGBoost: The maximum depth is 6, the Learning rate is 0.3, the learning goal is binary Logistic regression, and the evaluation indicator is AUC.

NCC is a single centroid classifier with no parameters.

DNN: Multiple perceptron model, the hidden layer is 100, weight optimization solver is *lbfgs*, and L2 penalty parameter is 0.0001.

**Supplementary Note 3: Evaluation Metrics**

In this paper, the MCC coefficient is chosen as the main evaluation index in the experiments. Matthews Correlation Coefficient integrates the evaluation of four indicators: true positive (TP), false positive (FP), true negative (TN), and false negative (FN). MCC is an evaluation index commonly used to measure the performance of classification models, especially for data sets with unbalanced sample sizes, MCC is a more effective evaluation metric (Chicco *et al.*, 2021).

$MCC=\frac{TP\times TN-FP\times FN}{\sqrt{(TP+FP)\times(TP+FN)\times(TN+FP)\times(TN+FN)}}$ (6)

In addition, we also used the following indicators for comprehensive evaluation.

AUC (Area Under the Curve): The ROC (Receiver Operating Characteristics) curve is plotted with TPR (True Positive Rate) against the FPR (False Positive Rate) where TPR is on the y-axis and FPR is on the x-axis.

Accuracy: the calculation formula is (TP+TN)/(TP+TN+FP+FN), which represents the proportion of samples in the total number of samples that the classifier predicts accurately.

Precision: the calculation formula is TP/(TP+FP), which represents the proportion of samples predicted to be positive by the classifier that are actually positive.

Recall: the calculation formula is TP/(TP+FN), which represents the proportion of correct model recognition in positive class samples.

F1-Score: the calculation formula is 2×Precision×Recall/(Precision+Recall), a comprehensive indicator that considers both accuracy and recall.

**Supplementary Note 4: Selection of critical features**

During the feature scanning stage, the feature sets involved in the top 10% of classifiers ranked by classification performance (MCC) were set as important feature sets. Ultimately, the importance of a feature is determined by the frequency of its occurrence in the important feature sets. Finally, the top ranked features (equal to the number of feature sets) are considered important features.

**Supplementary Note 5: Functional annotation**

In this study, we utilized Metascape (Zhou *et al.*, 2019) as the functional annotation tool. For the cancer detection task, the Ensembl IDs of all selected TSSs were employed as input for Metascape. Regarding the gene expression profiles used in cancer prognosis and drug sensitivity prediction, the gene symbols or gene IDs of the features were utilized for enrichment analysis.

However, as the corresponding gene symbols were unavailable for the methylation data in the drug prediction task, we chose Cistrome-GO as the enrichment analysis tool (Li *et al.*, 2019). Cistrome-GO allows users to input genome regions and obtain gene scores for subsequent gene ontology (GO) analysis and pathway enrichment. In our case, we used the genome regions of the selected DNA methylation probes as input. For all functional analysis, the significant GO Term Biological Process and KEGG Pathway were selected as the enriched functional gene sets.

**Supplementary Figures：**


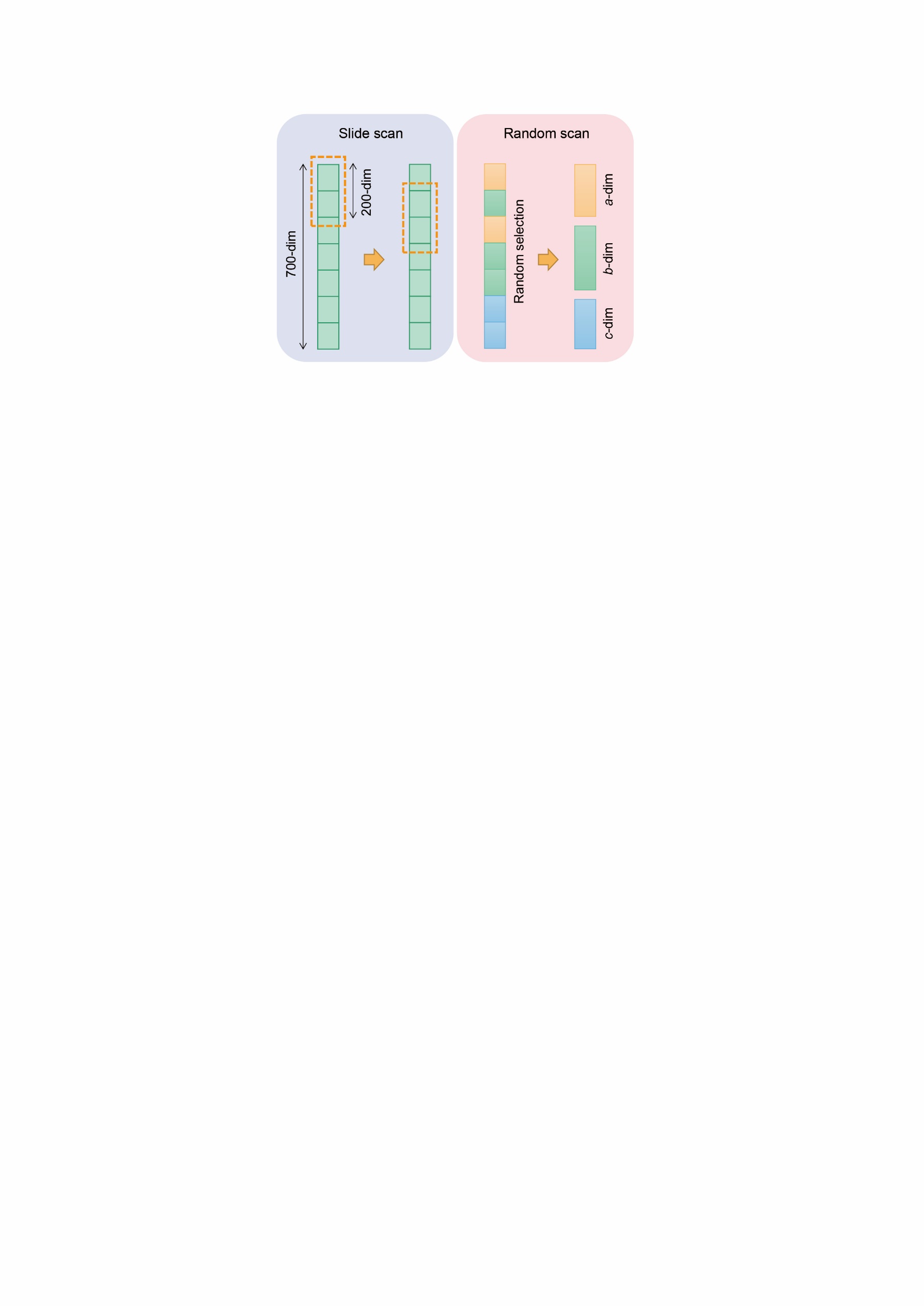


Supplementary Fig. 1 Comparison of feature scanning methods

**Supplementary Tables：**

Supplementary Table 1. Indicators for cross-validation of early diagnosis of cancer

| Methods | Accuracy | Precision | Recall | F1-score | AUC | MCC |
| --- | --- | --- | --- | --- | --- | --- |
| DC | 0.6927 | 0.7231 | 0.5214 | 0.6013 | 0.7326 | 0.3781 |
| RF | 0.5628 | 0.5371 | 0.2381 | 0.3228 | 0.5605 | 0.0840 |
| SVM | 0.6435 | 0.8231 | 0.2751 | 0.3998 | 0.7388 | 0.3059 |
| DF | 0.5660 | 0.5605 | 0.2128 | 0.2999 | 0.6041 | 0.0935 |
| XGBoost | 0.5394 | 0.4848 | 0.4179 | 0.4459 | 0.5347 | 0.0580 |
| NCC | 0.6846 | 0.7123 | 0.5119 | 0.5875 | 0.7324 | 0.3616 |
| DNN | 0.6533 | 0.6956 | 0.4186 | 0.5118 | 0.7026 | 0.2973 |

Supplementary Table 2. Indicators for independent validation of early diagnosis of cancer

| Methods | Accuracy | Precision | Recall | F1-score | AUC | MCC |
| --- | --- | --- | --- | --- | --- | --- |
| DC | 0.8035 | 0.2805 | 0.5370 | 0.3684 | 0.7168 | 0.2851 |
| RF | 0.8162 | 0.2151 | 0.2696 | 0.2388 | 0.6423 | 0.1373 |
| SVM | 0.8840 | 0.4231 | 0.2391 | 0.3056 | 0.7205 | 0.2596 |
| DF | 0.8510 | 0.2718 | 0.2391 | 0.2539 | 0.6568 | 0.1724 |
| XGBoost | 0.6473 | 0.1319 | 0.4130 | 0.2000 | 0.5903 | 0.0578 |
| NCC | 0.7958 | 0.2667 | 0.5217 | 0.3529 | 0.7143 | 0.2661 |
| DNN | 0.8165 | 0.2610 | 0.3804 | 0.3068 | 0.6760 | 0.2125 |

Supplementary Table 3. Enriched functional gene sets for early diagnosis of cancer (P<=0.01)

| Category | Pathway | -LogP |
| --- | --- | --- |
| GO Biological Processes | regionalization | 4.4023 |
| GO Biological Processes | negative regulation of satellite cell differentiation | 4.1962 |
| GO Biological Processes | pattern specification process | 4.0394 |
| GO Biological Processes | regulation of neuron death | 3.9741 |
| GO Biological Processes | negative regulation of skeletal muscle cell differentiation | 3.8065 |
| GO Biological Processes | B cell apoptotic process | 3.7637 |
| GO Biological Processes | cellular response to cytokine stimulus | 3.6815 |
| GO Biological Processes | basement membrane organization | 3.5316 |
| GO Biological Processes | heterophilic cell-cell adhesion via plasma membrane cell adhesion molecules | 3.4888 |
| GO Biological Processes | leukocyte cell-cell adhesion | 3.3364 |
| GO Biological Processes | leukocyte migration | 3.2672 |
| GO Biological Processes | cellular extravasation | 3.2607 |
| GO Biological Processes | leukocyte differentiation | 3.1119 |
| GO Biological Processes | cellular response to growth factor stimulus | 3.1033 |
| GO Biological Processes | negative regulation of cell differentiation | 3.0959 |
| GO Biological Processes | signal complex assembly | 3.0831 |
| GO Biological Processes | regulation of mesenchymal stem cell differentiation | 3.0831 |
| GO Biological Processes | cellular response to transforming growth factor beta stimulus | 3.0638 |
| GO Biological Processes | regulation of stem cell differentiation | 3.0563 |
| GO Biological Processes | response to growth factor | 3.0507 |
| GO Biological Processes | regulation of amino acid transmembrane transport | 3.0252 |
| GO Biological Processes | regulation of amino acid import across plasma membrane | 3.0252 |
| GO Biological Processes | positive regulation of actin filament bundle assembly | 3.0182 |
| GO Biological Processes | acetylcholine receptor signaling pathway | 2.9934 |
| GO Biological Processes | cell population proliferation | 2.9722 |
| GO Biological Processes | leukocyte migration involved in inflammatory response | 2.9313 |
| GO Biological Processes | response to transforming growth factor beta | 2.9034 |
| GO Biological Processes | cellular response to acetylcholine | 2.8664 |
| GO Biological Processes | leukocyte tethering or rolling | 2.8431 |
| GO Biological Processes | hemopoiesis | 2.7855 |
| GO Biological Processes | positive regulation of stem cell differentiation | 2.7600 |
| GO Biological Processes | positive regulation of stress fiber assembly | 2.7070 |
| GO Biological Processes | response to acetylcholine | 2.6926 |
| GO Biological Processes | leukocyte adhesion to vascular endothelial cell | 2.6816 |
| GO Biological Processes | mononuclear cell migration | 2.6795 |
| GO Biological Processes | postsynaptic signal transduction | 2.6385 |
| GO Biological Processes | positive regulation of phosphorylation | 2.4505 |
| GO Biological Processes | transforming growth factor beta receptor signaling pathway | 2.4303 |
| GO Biological Processes | myoblast migration | 2.4161 |
| GO Biological Processes | positive regulation of locomotion | 2.4092 |
| GO Biological Processes | mononuclear cell differentiation | 2.3936 |
| GO Biological Processes | regulation of lamellipodium assembly | 2.3932 |
| GO Biological Processes | positive regulation of leukocyte migration | 2.3589 |
| GO Biological Processes | muscle cell migration | 2.3450 |
| GO Biological Processes | T cell differentiation | 2.3195 |
| GO Biological Processes | lymphocyte differentiation | 2.2563 |
| GO Biological Processes | positive regulation of protein phosphorylation | 2.2493 |
| GO Biological Processes | positive regulation of lamellipodium assembly | 2.2310 |
| GO Biological Processes | positive regulation of cell migration | 2.2238 |
| GO Biological Processes | anterior/posterior pattern specification | 2.2146 |
| GO Biological Processes | positive T cell selection | 2.1775 |
| GO Biological Processes | entry into host | 2.1557 |
| GO Biological Processes | embryo development ending in birth or egg hatching | 2.1369 |
| GO Biological Processes | extracellular structure organization | 2.0504 |
| GO Biological Processes | positive regulation of neuron death | 2.0493 |
| GO Biological Processes | positive regulation of cell motility | 2.0012 |
| GO Biological Processes | positive regulation of cellular component biogenesis | 2.0007 |
| KEGG Pathway | Measles | 3.0670 |
| KEGG Pathway | Human immunodeficiency virus 1 infection | 2.9598 |
| KEGG Pathway | Pathways in cancer | 2.6037 |
| KEGG Pathway | Epstein-Barr virus infection | 2.2478 |

Supplementary Table 4. Indicators for cross-validation of cancer prognosis

| Methods | Accuracy | Precision | Recall | F1-score | AUC | MCC |
| --- | --- | --- | --- | --- | --- | --- |
| DC | 0.6569 | 0.4955 | 0.6103 | 0.5429 | 0.6788 | 0.2816 |
| RF | 0.6728 | 0.5662 | 0.0885 | 0.1483 | 0.6747 | 0.1230 |
| SVM | 0.6840 | 0.5588 | 0.3780 | 0.4414 | 0.7337 | 0.2482 |
| DF | 0.6721 | 0.5380 | 0.0691 | 0.1193 | 0.6939 | 0.1105 |
| XGBoost | 0.6474 | 0.4720 | 0.2646 | 0.3302 | 0.6345 | 0.1314 |
| NCC | 0.6427 | 0.4840 | 0.5982 | 0.5281 | 0.6837 | 0.2573 |
| DNN | 0.6888 | 0.5658 | 0.4058 | 0.4611 | 0.7115 | 0.2666 |

Supplementary Table 5. Indicators for independent validation of cancer prognosis

| Methods | Accuracy | Precision | Recall | F1-score | AUC | MCC |
| --- | --- | --- | --- | --- | --- | --- |
| DC | 0.7421 | 0.3236 | 0.6592 | 0.4336 | 0.7592 | 0.3226 |
| RF | 0.7596 | 0.2560 | 0.3079 | 0.2753 | 0.6233 | 0.1373 |
| SVM | 0.7224 | 0.2789 | 0.5395 | 0.3677 | 0.6976 | 0.2313 |
| DF | 0.8352 | 0.3768 | 0.1500 | 0.2139 | 0.6984 | 0.1596 |
| XGBoost | 0.7638 | 0.3000 | 0.4342 | 0.3548 | 0.6905 | 0.2217 |
| NCC | 0.7244 | 0.3025 | 0.6447 | 0.4118 | 0.7664 | 0.2932 |
| DNN | 0.7348 | 0.2874 | 0.5224 | 0.3694 | 0.6940 | 0.2362 |

Supplementary Table 6. Enrichment pathways for cancer prognosis (P<=0.01)

| Category | Pathway | -LogP |
| --- | --- | --- |
| GO Biological Processes | regulation of defense response | 8.8281 |
| GO Biological Processes | cell-cell adhesion | 7.2039 |
| GO Biological Processes | regulation of innate immune response | 6.0019 |
| GO Biological Processes | regulation of response to biotic stimulus | 5.5860 |
| GO Biological Processes | cell population proliferation | 5.3186 |
| GO Biological Processes | mammary gland morphogenesis | 5.1876 |
| GO Biological Processes | positive regulation of T cell tolerance induction | 5.0912 |
| GO Biological Processes | MAPK cascade | 5.0129 |
| GO Biological Processes | negative regulation of protein modification process | 4.9981 |
| GO Biological Processes | positive regulation of immune response | 4.9938 |
| GO Biological Processes | regulation of MAPK cascade | 4.9214 |
| GO Biological Processes | negative regulation of phosphate metabolic process | 4.9050 |
| GO Biological Processes | negative regulation of phosphorus metabolic process | 4.8903 |
| GO Biological Processes | glial cell development | 4.8243 |
| GO Biological Processes | gliogenesis | 4.8009 |
| GO Biological Processes | negative regulation of cell population proliferation | 4.7726 |
| GO Biological Processes | glial cell differentiation | 4.7687 |
| GO Biological Processes | regulation of inflammatory response | 4.7609 |
| GO Biological Processes | immune response-regulating signaling pathway | 4.6156 |
| GO Biological Processes | positive regulation of defense response | 4.5874 |
| GO Biological Processes | cell-cell adhesion via plasma-membrane adhesion molecules | 4.5816 |
| GO Biological Processes | positive regulation of tolerance induction | 4.5138 |
| GO Biological Processes | response to inorganic substance | 4.5133 |
| GO Biological Processes | regulation of T cell tolerance induction | 4.3597 |
| GO Biological Processes | retina homeostasis | 4.3233 |
| GO Biological Processes | negative regulation of intracellular signal transduction | 4.2603 |
| GO Biological Processes | gland development | 4.1977 |
| GO Biological Processes | positive regulation of response to external stimulus | 4.1547 |
| GO Biological Processes | regulation of cell activation | 4.0812 |
| GO Biological Processes | tissue homeostasis | 4.0556 |
| GO Biological Processes | anatomical structure homeostasis | 4.0556 |
| GO Biological Processes | mammary gland duct morphogenesis | 4.0218 |
| GO Biological Processes | immune response-activating signaling pathway | 3.9934 |
| GO Biological Processes | negative regulation of cell differentiation | 3.8741 |
| GO Biological Processes | regulation of leukocyte activation | 3.8491 |
| GO Biological Processes | multicellular organismal homeostasis | 3.8468 |
| GO Biological Processes | negative regulation of MAPK cascade | 3.6676 |
| GO Biological Processes | positive regulation of innate immune response | 3.6543 |
| GO Biological Processes | mammary gland development | 3.6496 |
| GO Biological Processes | negative regulation of catalytic activity | 3.5717 |
| GO Biological Processes | regulation of tolerance induction | 3.5679 |
| GO Biological Processes | activation of immune response | 3.5187 |
| GO Biological Processes | branching involved in mammary gland duct morphogenesis | 3.4817 |
| GO Biological Processes | immune response-regulating cell surface receptor signaling pathway | 3.4718 |
| GO Biological Processes | negative regulation of response to external stimulus | 3.4142 |
| GO Biological Processes | immune response-activating cell surface receptor signaling pathway | 3.3829 |
| GO Biological Processes | response to radiation | 3.3796 |
| GO Biological Processes | regulation of cell-cell adhesion | 3.3116 |
| GO Biological Processes | negative regulation of protein phosphorylation | 3.2881 |
| GO Biological Processes | negative regulation of phosphorylation | 3.2261 |
| GO Biological Processes | positive regulation of response to biotic stimulus | 3.2105 |
| GO Biological Processes | cellular response to UV | 3.0907 |
| GO Biological Processes | negative regulation of defense response | 3.0814 |
| GO Biological Processes | negative regulation of protein serine/threonine kinase activity | 3.0355 |
| GO Biological Processes | antigen receptor-mediated signaling pathway | 2.9944 |
| GO Biological Processes | behavior | 2.9740 |
| GO Biological Processes | negative regulation of T cell proliferation | 2.9498 |
| GO Biological Processes | mammary gland epithelial cell proliferation | 2.8681 |
| GO Biological Processes | gland morphogenesis | 2.8549 |
| GO Biological Processes | regulation of T cell activation | 2.8497 |
| GO Biological Processes | regulation of leukocyte cell-cell adhesion | 2.8385 |
| GO Biological Processes | regulation of lymphocyte activation | 2.7867 |
| GO Biological Processes | branch elongation of an epithelium | 2.7764 |
| GO Biological Processes | branching morphogenesis of an epithelial tube | 2.7355 |
| GO Biological Processes | regulation of lymphocyte proliferation | 2.7101 |
| GO Biological Processes | regulation of mononuclear cell proliferation | 2.6539 |
| GO Biological Processes | regulation of synaptic plasticity | 2.6333 |
| GO Biological Processes | mammary gland epithelium development | 2.6027 |
| GO Biological Processes | B cell receptor signaling pathway | 2.6027 |
| GO Biological Processes | cellular response to environmental stimulus | 2.5853 |
| GO Biological Processes | cellular response to abiotic stimulus | 2.5853 |
| GO Biological Processes | activation of innate immune response | 2.5451 |
| GO Biological Processes | tolerance induction | 2.5385 |
| GO Biological Processes | cellular response to radiation | 2.5167 |
| GO Biological Processes | negative regulation of protein kinase activity | 2.5159 |
| GO Biological Processes | negative regulation of lymphocyte proliferation | 2.5038 |
| GO Biological Processes | regulation of T cell proliferation | 2.5009 |
| GO Biological Processes | positive regulation of synaptic transmission | 2.4913 |
| GO Biological Processes | positive regulation of MAPK cascade | 2.4807 |
| GO Biological Processes | negative regulation of mononuclear cell proliferation | 2.4793 |
| GO Biological Processes | cellular response to light stimulus | 2.4746 |
| GO Biological Processes | innate immune response-activating signaling pathway | 2.4346 |
| GO Biological Processes | positive regulation of leukocyte activation | 2.4276 |
| GO Biological Processes | response to UV | 2.4219 |
| GO Biological Processes | positive regulation of inflammatory response | 2.4219 |
| GO Biological Processes | regulation of cellular response to stress | 2.3821 |
| GO Biological Processes | negative regulation of response to biotic stimulus | 2.3570 |
| GO Biological Processes | regulation of leukocyte proliferation | 2.3318 |
| GO Biological Processes | cell junction organization | 2.3270 |
| GO Biological Processes | intracellular receptor signaling pathway | 2.3222 |
| GO Biological Processes | negative regulation of leukocyte proliferation | 2.3175 |
| GO Biological Processes | hormone-mediated signaling pathway | 2.3010 |
| GO Biological Processes | cell-cell junction assembly | 2.2827 |
| GO Biological Processes | positive regulation of cell activation | 2.2617 |
| GO Biological Processes | negative regulation of T cell activation | 2.2467 |
| GO Biological Processes | liver development | 2.2467 |
| GO Biological Processes | developmental growth involved in morphogenesis | 2.2467 |
| GO Biological Processes | morphogenesis of a branching epithelium | 2.2431 |
| GO Biological Processes | pattern recognition receptor signaling pathway | 2.2317 |
| GO Biological Processes | negative regulation of kinase activity | 2.2233 |
| GO Biological Processes | response to light stimulus | 2.2197 |
| GO Biological Processes | cognition | 2.2197 |
| GO Biological Processes | oligodendrocyte development | 2.2187 |
| GO Biological Processes | homophilic cell adhesion via plasma membrane adhesion molecules | 2.2124 |
| GO Biological Processes | epithelial cell proliferation | 2.2124 |
| GO Biological Processes | hepaticobiliary system development | 2.1941 |
| GO Biological Processes | axis elongation | 2.1752 |
| GO Biological Processes | peripheral nervous system development | 2.1239 |
| GO Biological Processes | positive regulation of lymphocyte activation | 2.1172 |
| GO Biological Processes | response to hydrogen peroxide | 2.1110 |
| GO Biological Processes | regulation of JNK cascade | 2.1099 |
| GO Biological Processes | morphogenesis of a branching structure | 2.0945 |
| GO Biological Processes | regulation of peptidyl-serine phosphorylation | 2.0455 |
| GO Biological Processes | negative regulation of T cell mediated immunity | 2.0309 |
| GO Biological Processes | oligodendrocyte differentiation | 2.0308 |
| GO Biological Processes | cell-cell junction organization | 2.0247 |
| KEGG Pathway | Thyroid cancer | 4.5697 |
| KEGG Pathway | Small cell lung cancer | 3.8903 |
| KEGG Pathway | Pancreatic cancer | 3.6252 |
| KEGG Pathway | Chronic myeloid leukemia | 3.6252 |
| KEGG Pathway | FoxO signaling pathway | 3.5234 |
| KEGG Pathway | Endometrial cancer | 3.4537 |
| KEGG Pathway | MAPK signaling pathway | 3.4457 |
| KEGG Pathway | Tight junction | 3.3312 |
| KEGG Pathway | Colorectal cancer | 3.2949 |
| KEGG Pathway | Basal cell carcinoma | 3.2583 |
| KEGG Pathway | Leukocyte transendothelial migration | 3.2529 |
| KEGG Pathway | Non-small cell lung cancer | 2.9498 |
| KEGG Pathway | Melanoma | 2.9498 |
| KEGG Pathway | Transcriptional misregulation in cancer | 2.8883 |
| KEGG Pathway | Hepatitis B | 2.8602 |
| KEGG Pathway | Glioma | 2.8573 |
| KEGG Pathway | VEGF signaling pathway | 2.5700 |
| KEGG Pathway | Breast cancer | 2.5451 |
| KEGG Pathway | Pathways in cancer | 2.5347 |
| KEGG Pathway | Neurotrophin signaling pathway | 2.4746 |
| KEGG Pathway | Alcoholism | 2.4389 |
| KEGG Pathway | Hepatitis C | 2.3714 |
| KEGG Pathway | Endocrine resistance | 2.2740 |
| KEGG Pathway | Hepatocellular carcinoma | 2.1972 |
| KEGG Pathway | Progesterone-mediated oocyte maturation | 2.1904 |
| KEGG Pathway | p53 signaling pathway | 2.1729 |

Supplementary Table 7. Indicators for cross-validation of drug sensitivity prediction

| Methods | Accuracy | Precision | Recall | F1-score | AUC | MCC |
| --- | --- | --- | --- | --- | --- | --- |
| DC | 0.7564 | 0.2635 | 0.5478 | 0.3426 | 0.6900 | 0.2492 |
| RF | 0.8885 | 0.2898 | 0.0711 | 0.1034 | 0.7004 | 0.1116 |
| SVM | 0.8911 | 0.4380 | 0.1813 | 0.2377 | 0.7331 | 0.2255 |
| DF | 0.8889 | 0.2507 | 0.0666 | 0.0951 | 0.7229 | 0.1007 |
| XGBoost | 0.8857 | 0.3540 | 0.1234 | 0.1695 | 0.6876 | 0.1586 |
| NCC | 0.7393 | 0.2547 | 0.5729 | 0.3387 | 0.7160 | 0.2455 |
| DNN | 0.8802 | 0.4020 | 0.2159 | 0.2673 | 0.7260 | 0.2275 |

Supplementary Table 8. Enrichment pathways for drug sensitivity prediction (Gene expression data, P<=0.01)

| Category | Pathway | -LogP |
| --- | --- | --- |
| GO Biological Processes | cellular component morphogenesis | 7.0026 |
| GO Biological Processes | cell part morphogenesis | 6.7017 |
| GO Biological Processes | neuron projection development | 5.9656 |
| GO Biological Processes | regulation of transferase activity | 5.8951 |
| GO Biological Processes | plasma membrane bounded cell projection morphogenesis | 5.5720 |
| GO Biological Processes | cell projection morphogenesis | 5.4696 |
| GO Biological Processes | neuron projection morphogenesis | 5.2726 |
| GO Biological Processes | regulation of cell morphogenesis | 5.0709 |
| GO Biological Processes | regulation of axon extension | 4.9962 |
| GO Biological Processes | DNA-templated DNA replication | 4.9929 |
| GO Biological Processes | regulation of hormone levels | 4.9908 |
| GO Biological Processes | vesicle-mediated transport in synapse | 4.9650 |
| GO Biological Processes | chromosome organization | 4.9010 |
| GO Biological Processes | negative regulation of intracellular signal transduction | 4.8551 |
| GO Biological Processes | synaptic vesicle cycle | 4.7528 |
| GO Biological Processes | regulation of hydrolase activity | 4.5748 |
| GO Biological Processes | DNA replication | 4.4781 |
| GO Biological Processes | synapse organization | 4.4549 |
| GO Biological Processes | positive regulation of helicase activity | 4.4208 |
| GO Biological Processes | regulation of extent of cell growth | 4.1996 |
| GO Biological Processes | regulation of nervous system development | 4.0572 |
| GO Biological Processes | modulation of chemical synaptic transmission | 4.0210 |
| GO Biological Processes | regulation of proteolysis | 4.0111 |
| GO Biological Processes | regulation of trans-synaptic signaling | 4.0043 |
| GO Biological Processes | regulation of neurogenesis | 3.9479 |
| GO Biological Processes | regulation of cellular component size | 3.9449 |
| GO Biological Processes | regulation of kinase activity | 3.9156 |
| GO Biological Processes | hormone metabolic process | 3.7546 |
| GO Biological Processes | positive regulation of cell development | 3.7208 |
| GO Biological Processes | Wnt signaling pathway involved in midbrain dopaminergic neuron differentiation | 3.6772 |
| GO Biological Processes | positive regulation of transferase activity | 3.5373 |
| GO Biological Processes | regulation of helicase activity | 3.4924 |
| GO Biological Processes | regulation of protein kinase activity | 3.4614 |
| GO Biological Processes | cell morphogenesis involved in neuron differentiation | 3.4557 |
| GO Biological Processes | regulation of anatomical structure size | 3.3579 |
| GO Biological Processes | positive regulation of neurogenesis | 3.3370 |
| GO Biological Processes | regulation of cell projection organization | 3.3330 |
| GO Biological Processes | midbrain dopaminergic neuron differentiation | 3.3278 |
| GO Biological Processes | negative regulation of chemotaxis | 3.3229 |
| GO Biological Processes | regulation of cell size | 3.3030 |
| GO Biological Processes | regulation of synapse organization | 3.2931 |
| GO Biological Processes | axonogenesis | 3.2703 |
| GO Biological Processes | cell morphogenesis | 3.2342 |
| GO Biological Processes | canonical Wnt signaling pathway | 3.1897 |
| GO Biological Processes | cell junction organization | 3.1731 |
| GO Biological Processes | regulation of synapse structure or activity | 3.1647 |
| GO Biological Processes | positive regulation of nervous system development | 3.1605 |
| GO Biological Processes | regulation of cell junction assembly | 3.1272 |
| GO Biological Processes | regulation of axon extension involved in axon guidance | 3.1129 |
| GO Biological Processes | axon development | 3.0661 |
| GO Biological Processes | postsynapse organization | 3.0518 |
| GO Biological Processes | cell-cell signaling by wnt | 2.9845 |
| GO Biological Processes | Wnt signaling pathway | 2.9845 |
| GO Biological Processes | regulation of plasma membrane bounded cell projection organization | 2.9707 |
| GO Biological Processes | immune system development | 2.9528 |
| GO Biological Processes | positive regulation of hydrolase activity | 2.8401 |
| GO Biological Processes | positive regulation of proteolysis | 2.8347 |
| GO Biological Processes | signaling receptor ligand precursor processing | 2.7887 |
| GO Biological Processes | positive regulation of cell projection organization | 2.7552 |
| GO Biological Processes | endocytosis | 2.6982 |
| GO Biological Processes | negative regulation of axon extension involved in axon guidance | 2.6958 |
| GO Biological Processes | presynaptic endocytosis | 2.6741 |
| GO Biological Processes | mitotic cell cycle | 2.6627 |
| GO Biological Processes | regulation of neuron projection development | 2.6103 |
| GO Biological Processes | mitotic cell cycle process | 2.5988 |
| GO Biological Processes | mismatch repair | 2.4934 |
| GO Biological Processes | positive regulation of phosphate metabolic process | 2.4088 |
| GO Biological Processes | positive regulation of phosphorus metabolic process | 2.4088 |
| GO Biological Processes | import into cell | 2.3980 |
| GO Biological Processes | positive regulation of axonogenesis | 2.3820 |
| GO Biological Processes | synaptic vesicle recycling | 2.3757 |
| GO Biological Processes | DNA unwinding involved in DNA replication | 2.3479 |
| GO Biological Processes | regulation of synapse assembly | 2.3472 |
| GO Biological Processes | regulation of chemotaxis | 2.3392 |
| GO Biological Processes | dopaminergic neuron differentiation | 2.3145 |
| GO Biological Processes | negative regulation of DNA recombination | 2.2339 |
| GO Biological Processes | regulation of axonogenesis | 2.2234 |
| GO Biological Processes | peptide hormone processing | 2.2061 |
| GO Biological Processes | cellular response to retinoic acid | 2.1894 |
| GO Biological Processes | regulation of developmental growth | 2.1826 |
| GO Biological Processes | positive regulation of protein phosphorylation | 2.1668 |
| GO Biological Processes | forebrain regionalization | 2.1326 |
| GO Biological Processes | hematopoietic stem cell proliferation | 2.1326 |
| GO Biological Processes | positive regulation of phosphorylation | 2.1311 |
| GO Biological Processes | positive regulation of cell growth | 2.0978 |
| GO Biological Processes | regulation of cysteine-type endopeptidase activity involved in apoptotic process | 2.0978 |
| GO Biological Processes | regulation of peptidase activity | 2.0951 |
| GO Biological Processes | response to retinoic acid | 2.0737 |
| GO Biological Processes | positive regulation of kinase activity | 2.0726 |
| GO Biological Processes | synaptic vesicle endocytosis | 2.0710 |
| GO Biological Processes | DNA conformation change | 2.0395 |
| KEGG Pathway | Pathways in cancer | 7.5739 |
| KEGG Pathway | Basal cell carcinoma | 4.4208 |
| KEGG Pathway | Th17 cell differentiation | 4.2416 |
| KEGG Pathway | Breast cancer | 3.9608 |
| KEGG Pathway | Hepatocellular carcinoma | 3.8298 |
| KEGG Pathway | PD-L1 expression and PD-1 checkpoint pathway in cancer | 3.7817 |
| KEGG Pathway | Th1 and Th2 cell differentiation | 3.6527 |
| KEGG Pathway | Gastric cancer | 3.3654 |
| KEGG Pathway | T cell receptor signaling pathway | 3.1738 |
| KEGG Pathway | Wnt signaling pathway | 3.1668 |
| KEGG Pathway | Mismatch repair | 3.1021 |
| KEGG Pathway | Human T-cell leukemia virus 1 infection | 2.9287 |
| KEGG Pathway | Pathways of neurodegeneration - multiple diseases | 2.9032 |
| KEGG Pathway | Human papillomavirus infection | 2.7984 |
| KEGG Pathway | mTOR signaling pathway | 2.6887 |
| KEGG Pathway | Cell cycle | 2.6641 |
| KEGG Pathway | Alzheimer disease | 2.6443 |
| KEGG Pathway | Platinum drug resistance | 2.5613 |
| KEGG Pathway | Hematopoietic cell lineage | 2.2641 |
| KEGG Pathway | Melanogenesis | 2.2077 |
| KEGG Pathway | Primary immunodeficiency | 2.1052 |
| KEGG Pathway | Signaling pathways regulating pluripotency of stem cells | 2.1015 |
| KEGG Pathway | Proteoglycans in cancer | 2.0786 |

Supplementary Table 9. Enrichment pathways for drug sensitivity prediction (DNA methylation data, P<=0.01)

| Category | Pathway | -LogP |
| --- | --- | --- |
| GO Biological Processes | SRP-dependent cotranslational protein targeting to membrane, translocation | 6.5730 |
| GO Biological Processes | homophilic cell adhesion via plasma membrane adhesion molecules | 6.2053 |
| GO Biological Processes | neuron development | 5.8286 |
| GO Biological Processes | microtubule-based process | 5.5950 |
| GO Biological Processes | cell-cell adhesion via plasma-membrane adhesion molecules | 5.2986 |
| GO Biological Processes | positive regulation of biosynthetic process | 5.0167 |
| GO Biological Processes | positive regulation of nucleobase-containing compound metabolic process | 4.9771 |
| GO Biological Processes | protein transmembrane transport | 4.7970 |
| GO Biological Processes | positive regulation of transcription, DNA-templated | 4.5758 |
| GO Biological Processes | intracellular protein transmembrane transport | 4.3769 |
| GO Biological Processes | regulation of nervous system development | 4.1940 |
| GO Biological Processes | cell-cell adhesion | 4.0937 |
| GO Biological Processes | positive regulation of transcription by RNA polymerase II | 3.8998 |
| GO Biological Processes | neuron differentiation | 3.8776 |
| GO Biological Processes | multicellular organism growth | 3.8206 |
| GO Biological Processes | synapse assembly | 3.6492 |
| GO Biological Processes | system development | 3.6436 |
| GO Biological Processes | microtubule cytoskeleton organization | 3.5783 |
| GO Biological Processes | establishment of organelle localization | 3.5444 |
| GO Biological Processes | regulation of neuron differentiation | 3.5439 |
| GO Biological Processes | microtubule cytoskeleton organization involved in mitosis | 3.4910 |
| GO Biological Processes | positive regulation of nervous system development | 3.4252 |
| GO Biological Processes | regulation of cell development | 3.4053 |
| GO Biological Processes | regulation of multicellular organismal development | 3.3537 |
| GO Biological Processes | cell cycle process | 3.1872 |
| GO Biological Processes | ribosome biogenesis | 3.1530 |
| GO Biological Processes | peptide catabolic process | 3.1216 |
| GO Biological Processes | glandular epithelial cell differentiation | 3.0709 |
| GO Biological Processes | animal organ development | 3.0377 |
| GO Biological Processes | synapse organization | 3.0339 |
| GO Biological Processes | regulation of neuron projection development | 3.0099 |
| GO Biological Processes | myeloid leukocyte mediated immunity | 2.9181 |
| GO Biological Processes | regulation of cell differentiation | 2.9113 |
| GO Biological Processes | positive regulation of gene expression | 2.8524 |
| GO Biological Processes | cell development | 2.8470 |
| GO Biological Processes | mitotic cell cycle process | 2.8384 |
| GO Biological Processes | positive regulation of triglyceride biosynthetic process | 2.8291 |
| GO Biological Processes | glutathione catabolic process | 2.8227 |
| GO Biological Processes | embryonic skeletal system morphogenesis | 2.7921 |
| GO Biological Processes | biological adhesion | 2.7685 |
| GO Biological Processes | negative regulation of response to DNA damage stimulus | 2.7313 |
| GO Biological Processes | branching involved in ureteric bud morphogenesis | 2.7138 |
| GO Biological Processes | regulation of cellular response to stress | 2.6998 |
| GO Biological Processes | purine nucleoside monophosphate metabolic process | 2.6839 |
| GO Biological Processes | regulation of cellular component biogenesis | 2.6661 |
| GO Biological Processes | embryonic organ morphogenesis | 2.6266 |
| GO Biological Processes | stem cell proliferation | 2.6219 |
| GO Biological Processes | response to morphine | 2.6172 |
| GO Biological Processes | positive regulation of triglyceride metabolic process | 2.5935 |
| GO Biological Processes | SMAD protein signal transduction | 2.5634 |
| GO Biological Processes | positive regulation of centrosome cycle | 2.5626 |
| GO Biological Processes | growth | 2.5444 |
| GO Biological Processes | regulation of nuclear division | 2.5026 |
| GO Biological Processes | protein-containing complex subunit organization | 2.4857 |
| GO Biological Processes | positive regulation of pathway-restricted SMAD protein phosphorylation | 2.4818 |
| GO Biological Processes | positive regulation of cellular component organization | 2.4760 |
| GO Biological Processes | negative regulation of cellular component organization | 2.4406 |
| GO Biological Processes | microtubule-based movement | 2.4252 |
| GO Biological Processes | cell differentiation | 2.3983 |
| GO Biological Processes | metaphase plate congression | 2.3904 |
| GO Biological Processes | negative regulation of neuron death | 2.3851 |
| GO Biological Processes | response to drug | 2.3763 |
| GO Biological Processes | regulation of cell growth | 2.3633 |
| GO Biological Processes | neurogenesis | 2.3551 |
| GO Biological Processes | negative regulation of neuron differentiation | 2.3486 |
| GO Biological Processes | positive regulation of synapse assembly | 2.3417 |
| GO Biological Processes | negative regulation of cell development | 2.3231 |
| GO Biological Processes | adult behavior | 2.2814 |
| GO Biological Processes | regulation of pathway-restricted SMAD protein phosphorylation | 2.2740 |
| GO Biological Processes | glutathione metabolic process | 2.2658 |
| GO Biological Processes | regulation of response to DNA damage stimulus | 2.2629 |
| GO Biological Processes | skeletal system morphogenesis | 2.2410 |
| GO Biological Processes | telomeric loop disassembly | 2.2391 |
| GO Biological Processes | leukocyte mediated immunity | 2.2328 |
| GO Biological Processes | formation of cytoplasmic translation initiation complex | 2.2257 |
| GO Biological Processes | photoreceptor cell development | 2.2222 |
| GO Biological Processes | ribonucleoside monophosphate metabolic process | 2.2092 |
| GO Biological Processes | negative regulation of nervous system development | 2.1959 |
| GO Biological Processes | negative regulation of cell differentiation | 2.1875 |
| GO Biological Processes | protein localization to endoplasmic reticulum | 2.1799 |
| GO Biological Processes | leukotriene biosynthetic process | 2.1731 |
| GO Biological Processes | positive regulation of transmembrane receptor protein serine/threonine kinase signaling pathway | 2.1623 |
| GO Biological Processes | cytoskeleton organization | 2.1620 |
| GO Biological Processes | positive regulation of binding | 2.1581 |
| GO Biological Processes | negative regulation of fat cell differentiation | 2.1529 |
| GO Biological Processes | regulation of organelle organization | 2.1475 |
| GO Biological Processes | regulation of protein complex assembly | 2.1160 |
| GO Biological Processes | ether metabolic process | 2.1150 |
| GO Biological Processes | regulation of neuron apoptotic process | 2.0973 |
| GO Biological Processes | negative regulation of DNA recombination | 2.0833 |
| GO Biological Processes | regulation of supramolecular fiber organization | 2.0762 |
| GO Biological Processes | positive regulation of organelle organization | 2.0707 |
| GO Biological Processes | GMP metabolic process | 2.0680 |
| GO Biological Processes | positive regulation of establishment of protein localization to mitochondrion | 2.0612 |
| GO Biological Processes | establishment of spindle orientation | 2.0549 |
| GO Biological Processes | negative regulation of cell junction assembly | 2.0479 |
| GO Biological Processes | negative regulation of RNA splicing | 2.0324 |
| GO Biological Processes | organelle fission | 2.0311 |
| GO Biological Processes | cell junction organization | 2.0282 |
| GO Biological Processes | positive regulation of developmental process | 2.0198 |
| GO Biological Processes | negative regulation of neuron projection development | 2.0115 |
| GO Biological Processes | negative regulation of cell projection organization | 2.0064 |
| GO Biological Processes | chromatin remodeling | 2.0035 |
| GO Biological Processes | regulation of neuron death | 2.0006 |
| KEGG Pathway | Protein export | 3.4672 |
| KEGG Pathway | Hippo signaling pathway | 2.9492 |
| KEGG Pathway | Rap1 signaling pathway | 2.6703 |
| KEGG Pathway | Legionellosis | 2.0557 |

Supplementary Table 10. Comparison between random scanning and sliding window scanning (early diagnosis of cancer)

| Methods | Accuracy | Precision | Recall | F1-score | AUC | MCC |
| --- | --- | --- | --- | --- | --- | --- |
| random scanning (CV) | 0.6927 | 0.7231 | 0.5214 | 0.6013 | 0.7327 | 0.3781 |
| sliding window scanning (CV) | 0.6854 | 0.7103 | 0.5166 | 0.5929 | 0.7288 | 0.3629 |
| random scanning (IV) | 0.8035 | 0.2805 | 0.5370 | 0.3684 | 0.7164 | 0.2851 |
| sliding window scanning (IV) | 0.7926 | 0.2555 | 0.4913 | 0.3360 | 0.6942 | 0.2446 |

Supplementary Table 11. Comparison between NCC and other two strategies (RF and MM, early diagnosis of cancer)

| Methods | Accuracy | Precision | Recall | F1-score | AUC | MCC |
| --- | --- | --- | --- | --- | --- | --- |
| DeepCentroid (CV) | 0.6927 | 0.7231 | 0.5214 | 0.6013 | 0.7327 | 0.3781 |
| Integrated RF (CV) | 0.5702 | 0.5394 | 0.3298 | 0.4035 | 0.6068 | 0.1088 |
| Integrated MM (CV) | 0.6711 | 0.7008 | 0.4796 | 0.5637 | 0.7112 | 0.3335 |
| DeepCentroid (IV) | 0.8035 | 0.2805 | 0.5370 | 0.3684 | 0.7164 | 0.2851 |
| Integrated RF (IV) | 0.8253 | 0.2685 | 0.3696 | 0.3110 | 0.6301 | 0.2175 |
| Integrated MM (IV) | 0.8009 | 0.2636 | 0.4761 | 0.3384 | 0.7002 | 0.2475 |

Supplementary Table 12. Comparison between random scanning and sliding window scanning (cancer prognosis)

| Methods | Accuracy | Precision | Recall | F1-score | AUC | MCC |
| --- | --- | --- | --- | --- | --- | --- |
| random scanning (CV) | 0.6569 | 0.4955 | 0.6103 | 0.5429 | 0.6788 | 0.2816 |
| sliding window scanning (CV) | 0.6459 | 0.4831 | 0.6007 | 0.5309 | 0.6870 | 0.2609 |
| random scanning (IV) | 0.7421 | 0.3236 | 0.6592 | 0.4336 | 0.7490 | 0.3226 |
| sliding window scanning (IV) | 0.7057 | 0.2981 | 0.7105 | 0.4195 | 0.7640 | 0.3097 |

Supplementary Table 13. Comparison between NCC and other two strategies (RF and MM, cancer prognosis)

| Methods | Accuracy | Precision | Recall | F1-score | AUC | MCC |
| --- | --- | --- | --- | --- | --- | --- |
| DeepCentroid (CV) | 0.6569 | 0.4955 | 0.6103 | 0.5429 | 0.6788 | 0.2816 |
| Integrated RF (CV) | 0.6717 | 0.5901 | 0.1572 | 0.2358 | 0.6978 | 0.1532 |
| Integrated MM (CV) | 0.6826 | 0.5478 | 0.4073 | 0.4605 | 0.7099 | 0.2532 |
| DeepCentroid (IV) | 0.7421 | 0.3236 | 0.6592 | 0.4336 | 0.7490 | 0.3226 |
| Integrated RF (IV) | 0.8043 | 0.3317 | 0.3026 | 0.3152 | 0.7315 | 0.2026 |
| Integrated MM (IV) | 0.7429 | 0.3033 | 0.5408 | 0.3861 | 0.7149 | 0.2583 |

Supplementary Table 14. Comparison between random scanning and sliding window scanning (drug sensitivity prediction)

| Methods | Accuracy | Precision | Recall | F1-score | AUC | MCC |
| --- | --- | --- | --- | --- | --- | --- |
| random scanning (CV) | 0.7571 | 0.2635 | 0.5460 | 0.3425 | 0.6896 | 0.2489 |
| sliding window scanning (CV) | 0.7493 | 0.2592 | 0.5616 | 0.3416 | 0.7063 | 0.2487 |

Supplementary Table 15. Comparison between NCC and other two strategies (RF and MM, drug sensitivity prediction)

| Methods | Accuracy | Precision | Recall | F1-score | AUC | MCC |
| --- | --- | --- | --- | --- | --- | --- |
| DeepCentroid (CV) | 0.7571 | 0.2635 | 0.5460 | 0.3425 | 0.6896 | 0.2489 |
| Integrated RF (CV) | 0.8840 | 0.3670 | 0.2002 | 0.2398 | 0.7438 | 0.2108 |
| Integrated MM (CV) | 0.8871 | 0.4144 | 0.1726 | 0.2253 | 0.7364 | 0.2078 |

**Supplementary References**

Chicco D. *et al.* (2021) The Matthews correlation coefficient (MCC) is more reliable than balanced accuracy, bookmaker informedness, and markedness in two-class confusion matrix evaluation. *BioData mining*, 14(1): 1-22.

Desmedt C. *et al.* (2007) Strong time dependence of the 76-gene prognostic signature for node-negative breast cancer patients in the TRANSBIG multicenter independent validation series. *Clinical cancer research*, 13(11): 3207-3214.

Iorio F. *et al.* (2016) A landscape of pharmacogenomic interactions in cancer. *Cell*, 166(3): 740-754.

Li S. *et al.* (2019) Cistrome-GO: a web server for functional enrichment analysis of transcription factor ChIP-seq peaks. *Nucleic acids research*, 47(W1): W206-W211.

Mathios D. *et al.* (2021) Detection and characterization of lung cancer using cell-free DNA fragmentomes. *Nature communications*, 12(1): 5060.

Schmidt M. *et al.* (2008) The humoral immune system has a key prognostic impact in node-negative breast cancer. *Cancer research*, 68(13): 5405-5413.

Ulz P. *et al.* (2016) Inferring expressed genes by whole-genome sequencing of plasma DNA. *Nature genetics*, 48(10): 1273-1278.

Wang Y. *et al.* (2005) Gene-expression profiles to predict distant metastasis of lymph-node-negative primary breast cancer. *The Lancet*, 365(9460): 671-679.

Zhang Y. *et al.* (2009) The 76-gene signature defines high-risk patients that benefit from adjuvant tamoxifen therapy. *Breast cancer research and treatment*, 116: 303-309.

Zhou Y. *et al.* (2019) Metascape provides a biologist-oriented resource for the analysis of systems-level datasets. *Nature communications*, 10(1): 1523.
